# Association of Sleep Patterns and Respiratory Disturbance Index with Physiological Parameters in Pediatric Patients with Self-Perceived Short Stature

**DOI:** 10.3390/diagnostics14151675

**Published:** 2024-08-02

**Authors:** Jing-Yang Huang, Pei-Lun Liao, Hua-Pin Chang, Pen-Hua Su

**Affiliations:** 1 Department of Medical Research, Chung Shan Medical University Hospital, Taichung 40201, Taiwan; cshe961@csh.org.tw (J.-Y.H.); cshe1200@csh.org.tw (P.-L.L.); 2 Institute of Medicine, Chung Shan Medical University, Taichung 40201, Taiwan; 3 Department of Nursing, Asia University, Taichung 41354, Taiwan; 4 Department of Pediatrics, Chung Shan Medical University Hospital, Taichung 40201, Taiwan; 5 School of Medicine, Chung Shan Medical University, Taichung 40201, Taiwan

**Keywords:** children, sleep, precocious puberty, home portable sleep detection device

## Abstract

Objective: To investigate the relationships of sleep patterns and respiratory disturbance index (RDI) with key physiological parameters (height, body mass index (BMI), bone age (BA), and IGF-1 levels) in children aged 6 to 16 years with self-perceived short stature. Methods: For this cross-sectional study, conducted from October 2019 to November 2021, 238 children aged 6 to 16 years with self-perceived short stature were enrolled. The primary outcomes of sleep patterns and the RDI were non-invasively collected at home using the LARGAN Health AI-Tech Sleep Apnea and Sleep Quality Examination System, which operates based on polygraphy. Additionally, various physiological parameters, including height, BMI, bone age, and IGF-1 levels, were measured to assess their associations with sleep patterns and RDI. Results: Significant age-related reductions were observed in both the total and deep sleep durations. Children aged 6–9 years averaged 8.5 ± 1.0 h of total sleep, which decreased to 8.1 ± 1.1 h in ages 10–11 and further to 7.5 ± 0.9 h in ages 12–16 (*p* < 0.0001). Deep sleep followed a similar pattern, decreasing from 4.4 ± 1.1 h in the youngest group to 3.3 ± 1.0 h in the oldest (*p* < 0.0001). Notably, girls experienced significantly longer deep sleep than boys, averaging 4.0 ± 1.2 h compared to 3.6 ± 1.2 h (*p* = 0.0153). In a multivariable regression analysis, age (beta = 4.89, *p* < 0.0001) and RDI (beta = −0.54, *p* = 0.0022) were significantly associated with body height. Age and deep sleep duration (beta = −0.02, *p* = 0.0371) were significantly associated with BMI. Conclusions: The results demonstrate significant age-related decreases in the total and deep sleep duration among children with self-perceived short stature, along with a notable association between RDI and body height and an association between deep sleep duration and BMI. These findings suggest that sleep disturbances in pediatric endocrine patients are intricately linked with physiological growth parameters. The identified correlations underline the importance of monitoring sleep patterns in this demographic to better understand the impact of endocrine disorders on developmental health. Further research is needed to explore interventions that could alleviate these sleep disturbances, thereby potentially improving outcomes for the affected children.

## 1. Introduction

Sleep is a multifaceted phenomenon, encompassing both objective and subjective experiences. It is distinguished by various stages, such as rapid eye movement (REM) sleep and non-rapid eye movement (NREM) sleep [1]. NREM sleep is further divided into three stages: N1, N2, and N3. These classifications are determined according to electroencephalographic (EEG) criteria and the depth of sleep [2]. Sleep initiation typically begins with the NREM phase, progressing through the deeper NREM stages—N2 and N3—before transitioning into the first episode of REM sleep approximately 80 to 100 min later. After this initial REM episode, there is an alternation between NREM and REM sleep, following a cycle duration of about 90 min. In the early part of the night, the initial NREM cycles are predominantly in the N3 stage of deep NREM sleep [3,4]. As the night progresses, the duration of REM sleep episodes increases, reflecting the body’s changing needs for restorative sleep early at night and, in the later cycles, the increased importance of REM sleep, which is associated with dreaming and cognitive processing.

While parental questionnaires have offered initial insights into children’s sleep, questionnaires have limited accuracy across various sleep measures, necessitating the exploration of more precise methods [5]. Polysomnography (PSG), the gold standard, provides comprehensive physiological data, including electroencephalogram (EEG), eye movement graph (EOG), electromyography (EMG), and electrocardiogram (ECG) data in addition to information about blood oxygen saturation (SaO2), pulse, airflow, etc. [6]. However, PSG is often not readily available in clinical practice due to the need for a specialized laboratory setting and the high costs that are involved, particularly for pediatric populations. These limitations underscore the importance of accessible and cost-effective alternatives such as portable sleep devices. A recent study that utilized the ultrasound features of the diaphragm to screen for obstructive sleep apnea (OSA) demonstrated the utility of diaphragmatic ultrasound in predicting OSA with high sensitivity and specificity, suggesting its value as a promising screening tool [7]. Due to advancements in home sleep-monitoring devices, promising and cost-effective alternatives such as actigraphy and cardiovascular measurements can now be offered, demonstrating impressive accuracy. For instance, actigraphy boasts high accuracy in measuring sleep efficiency [8], while ECG recordings can be used to assess both sleep efficiency and the apnea–hypopnea index (AHI) [9]. The AHI is crucial for determining the severity of obstructive sleep apnea (OSA) in children [10] for providing essential diagnostic and therapeutic guidance.

According to the consensus statement from the American Academy of Sleep Medicine (AASM), it is recommended that children aged 6 to 12 years sleep for 9 to 12 h per 24 h on a regular basis, while the recommended amount of sleep to promote optimal health for teenagers aged 13 to 18 years is 8 to 10 h per 24 h [11]. Sleep quality is closely associated with various aspects of cognitive and emotional functioning in children and adolescents, including attention, behavior, learning, memory, and emotional regulation [12,13,14]. Additionally, in young individuals, insufficient sleep has been linked to an increased risk of accidents, injuries, hypertension, obesity, diabetes, and depression [14,15]. Among teenagers, sleep problems, particularly insomnia, have been specifically associated with an increased likelihood of engaging in self-harm [16,17]. The consequences of sleep deprivation, which are correlated with growth hormone and ghrelin levels, significantly influence growth and obesity [18]. This is a crucial risk factor for OSA, underscoring the need for targeted sleep interventions in this population.

A bidirectional relationship exists between sleep and endocrine activity, affecting sleep architecture and contributing to sleep disorders. Key hormones like GHRH, CRH, ghrelin, and prolactin modulate sleep stages and duration, with implications for aging, depression, and other conditions [19]. Understanding the complex interplay between sleep and hormones can lead to the development of targeted interventions for sleep disturbances and improved overall health. The amount of sleep needed by individuals is subject to variability and can be influenced by a combination of genetic, behavioral, medical, and environmental factors [20]. The sleep patterns of children and adolescents differ significantly from those of adults. Children require more sleep, with variations in sleep architecture and duration as they age. The existing studies in adults have shown a relationship between sleep parameters and endocrine disorders, highlighting the impact of sleep on hormonal regulation and metabolic functions. Few studies have, however, investigated the distribution of sleep patterns and respiratory disturbance index (RDI) in children with self-perceived short stature, particularly regarding the relationship between RDI and their height, obesity, and development-related biomarkers. Therefore, this study was conducted on children who visited an endocrine clinic in central Taiwan due to self-perceived short stature, aiming to investigate the distribution of their sleep patterns and respiratory disturbance index (RDI), and to explore the relationship between sleep patterns and their height, body mass index (BMI), bone age, and insulin-like growth factor 1 (IGF-1). The findings of this study may inform the development of potential recommendations for sleep management in these patients.

## 2. Methods

### 2.1. Study Design and Participants

For this cross-sectional study, conducted between October 2019 and November 2021, children aged 6 to 16 years with self-perceived short stature were recruited from the pediatric endocrinology outpatient department at Chung Shan Medical University Hospital (CSMUH). We performed a sample size calculation using the G*Power v3.1.9.7 software with the test family set as F tests; the statistical test was based on the linear multiple regression: fixed model, R^2^ deviation from zero, with an effect size (f) of 0.15, an alpha error of 0.05, a power of 0.80, and 36 predictors. The required sample size was calculated to be 203. Therefore, we aimed to collect data from 203 participants, and 238 children were ultimately included in the study to account for any potential dropouts or incomplete data. These children were referred to the endocrine center for the evaluation of growth concerns, including short stature, delayed growth velocity, and other related symptoms. The exact diagnoses included conditions such as growth hormone deficiency, Turner syndrome, and hypothyroidism. Participation involved written informed consent from parents and children and willingness to use an actigraphy device (portable sleep detection device) at home for two days (one night). Children with severe respiratory or cardiac conditions and those using positive pressure therapy, oxygen therapy, or medications affecting sleep were excluded. Of the 239 initially enrolled children, one with an incomplete sleep examination was excluded, resulting in a final sample of 238 participants for analysis. The study received ethical approval from the Institutional Review Board of CSMUH (approval number CS1-20209).

### 2.2. Sleep Apnea and Sleep Quality Examination

We utilized the LARGAN Health AI-TECH Co., LTD, Taichung City, Taiwan. Sleep Apnea and Sleep Quality Examination System. This medical device has received approval from the Taiwan Ministry of Health and Welfare (MOHW) (License Number: MOHW Medical Devices Manufacturing No. 007003; license valid until 4 January 2026). The LARGAN Health AI-Tech Sleep Apnea and Sleep Quality Examination System is a sophisticated software solution that leverages inputs from ECG and accelerometer (ACC) signals recorded during sleep by a portable ECG Holter monitor. The ACC measures physical motion, providing data on patient movement and position during sleep. It utilizes the ECG signals to calculate the sinus heart rate and detect breathing-related movements during sleep. Through a comprehensive analysis of the sinus heart rate and sleep breathing patterns, the system evaluates the subject’s sleep quality, identifies disruptions in sleep breathing, and measures both heart rate and heart rate variability (HRV). Additionally, the ACC signal is analyzed to assess behaviors such as lying down and waking up during the night. This product is designed for user-friendly operation, causes minimal sleep disturbance, and is suitable for home testing. The PhysioNet Apnea-ECG Database was utilized to test the validity of the LARGAN Health AI-Tech Sleep Apnea and Sleep Quality Examination System. This database comprises 70 polygraphy recordings, which are collected using Sandman® SD20 PSG System or equivalent polygraph equipment. Certified sleep experts have manually scored all the recordings. Apneic events were identified, and the AHI was calculated for each recording. The results of the sleep apnea analysis by this device were subjected to comparison with manual scoring by sleep experts. The device demonstrated a sensitivity of 95.0% (95% CI: 83.5 to 98.6), a specificity of 90.0% (95% CI: 74.4 to 96.5), and positive predictive values of 92.7%.

We provided the Largan Health AI-Tech home equipment instructions, depicted in Supplementary Figure S1. Concurrently, one-on-one guidance was offered by a nurse, fully trained in operating the Largan Health AI-Tech home equipment, to the subject’s mother or father regarding the correct usage. A contact phone number was also made available to the parents to resolve any encountered difficulties. The participants’ data were transmitted, via the network, to cloud storage for analysis. To be included in this study, the subjects must have a valid ECG data percentage of over 80%. The parents supplied information regarding their children, including bedtime, wake-up time, out-of-bed time, height, and weight. Upon the completion of the sleep test, the parents were instructed to return the device to the hospital and hand it over to the research nurse.

### 2.3. Study Variables

In this study, we collected data related to demographic variables (age and gender), body height, body weight, bone age, IGF-1 levels, the presence or absence of enlarged adenoid tonsil in the posterior nasopharynx, and the growth hormone therapy. The sleep apnea examination delivered comprehensive results pertinent to sleep quality, including time in bed, total sleep time, and sleep onset time, providing insights into the duration and onset of sleep. Sleep staging was conducted, quantifying periods of deep sleep, light sleep, REM sleep, and periods of wakefulness, all measured in minutes. The examination also involved the meticulous documentation of sleep breathing disruptions, indicated by the number of sleep breathing disorder events and quantified as the respiratory disturbance index (RDI). Furthermore, it records the duration of nocturnal awakenings, offering a complete overview of the subject’s sleep pattern.

BMI is calculated as the individual’s body weight in kilograms divided by the square of their height in meters. To account for the effects of age or sex, variables such as body height, BMI, and IGF-1 levels are standardized using Z-scores. The Z-score is computed as the difference in the measured value in the individual from the average value of a similar age and sex population, divided by the standard deviation. To compare differences in sleep patterns and the RDI, the participants are categorized into age groups of 6–9, 10–11, and 12–16 years; height Z-scores as ≤−1, between −1 and +1, and ≥1; and BMI Z-scores as ≤−1, between −1 and +1, and ≥1. We calculated bone age (BA) using radiographs of the left hand and determined the ratio of bone age to chronological age (BA/CA ratio). The BA/CA ratios were classified as ≤1.2 and >1.2. According to [21], progressive central precocious puberty is more likely when BA/CA exceeds 1.2. Similarly, IGF-1 Z-scores were categorized as <0 and ≥0. For the assessment of obstructive sleep apnea (OSA) severity, the RDI ranges were categorized according to the diagnostic criteria defined by the American Academy of Sleep Medicine, which defines pediatric OSA diagnosis as RDI  ≥  1. In the present study, OSA was classified as mild (1  ≤  RDI  <  5) or moderate/severe (RDI  ≥  5) [22].

### 2.4. Statistical Methods

The descriptive statistics for sleep patterns and sleep apnea examination results of this study, including the duration (in minutes) of total sleep time, light sleep, deep sleep, REM sleep, and the RDI, are expressed as mean ± standard deviation (SD). The normality of the data, which was confirmed using the Shapiro–Wilk test, supports the use of parametric tests, such as the independent t-test and ANOVA, for testing the differences in sleep patterns and sleep apnea examination results between categorical groups. Furthermore, the comparison was stratified according to age groups (6–9, 10–11, and 12–16 years old). Linear regression was employed to explore the associations of age, sex, sleep patterns, and sleep apnea with body height and BMI. For linear regression analysis, R-squared (R^2^) was used to assess the goodness of fit of a model, where a higher R^2^ value indicates better fit and suggests that the model can more accurately capture the observed outcomes. Data analysis in this study was conducted using the SAS software (SAS software, Version 9.4. SAS Institute Inc., Cary, NC, USA.). Statistical analyses were performed using two-tailed tests, with a *p*-value of <0.05 considered to indicate statistical significance.

## 3. Results

### 3.1. Characteristics of Study Participations

Table 1 presents the demographic, body measurements, sleep patterns, and sleep apnea characteristics of the study participants. For the study, 238 children were included: 23 (9.7%) were measured between January and March, 13 (5.5%) were measured between April and June, 83 (34.9%) were measured between July and September, and 119 (50.0%) were measured between October and December. Of the children, 204 (85.7%) were measured on weekend nights (Friday and Saturday), while 34 (14.3%) were measured on weeknights (Sunday to Thursday). The majority, 57.6%, were boys (137 out of 238). The age distribution among the participants was as follows: 30.3% (72 out of 238) were aged 6 to 9 years, 28.6% (68 out of 238) were between 10 and 11 years, and the largest age group comprised children aged 12 to 16 years, representing 41.2% (98 out of 238) of the sample. The mean height of the participants was 140.55 cm, with an SD of ±14.59 cm, and the average height Z-score was −0.85 ± 1.02. The BMI averaged 35.84 ± 12.53, with an average BMI Z-score of −0.21 ± 1.26. The mean BA/CA ratio was 1.00 ± 0.15. The mean IGF-1 level was 234.91 ± 108.72, with a mean Z-score of 0.00 ± 0.96. Clinically, 36.6% of the children displayed enlarged adenoid tonsils in the posterior nasopharynx. Regarding sleep patterns, the average sleep duration was 7.99 ± 1.08 h, with the light sleep phase averaging 2.48 ± 0.85 h, the deep sleep phase 3.79 ± 1.18 h, and the REM sleep phase 1.73 ± 0.68 h. The mean RDI was 3.51 ± 2.87. According to the relevant criteria, 19 (8.0%) children did not have OSA (defined as RDI < 1), 184 (77.3%) had mild OSA (1  ≤  RDI < 5), and 35 (14.7%) had moderate or severe OSA (RDI  ≥  5). Additionally, 99 (41.6%) of the children experienced sleep latency of more than 30 min, and a total of 68 (28.6%) children received growth hormone therapy.

### 3.2. Differences in States of Sleep among Children 

Table 2 presents the distribution of sleep patterns and respiratory disturbance index across the different groups categorized by seasonality, weekend night, age, sex, height Z-score, BMI Z-score, BA/CA ratios, IGF-1 levels, growth hormone therapy, and RDI levels. The children measured during summer (defined as April–September) had significantly longer REM duration (mean ± SD: 1.87 ± 0.72 h) compared to those measured during winter (defined as October–March) (1.64 ± 0.64 h); however, there were no significant differences in the total sleep time and RDI between summer and winter. There were no significant differences in sleep patterns and RDI between weekend nights and weeknights. The age group of 12–16 years exhibited the shortest total sleep duration (mean ± SD: 7.53 ± 0.93 h) and the smallest amount of deep sleep (3.34 ± 1.03 h). A decline in REM sleep duration with increasing age was noted in the 12–16 age group, though these differences were not statistically significant (*p* = 0.1484). The 6–9 age group displayed a significantly higher occurrence of disturbance according to the RDI (4.10 ± 3.04), and the difference compared to other age groups was statistically significant (*p* = 0.0457). A statistically significant difference in deep sleep duration was observed between girls and boys, with girls having longer deep sleep (4.00 ± 1.15 h) than boys (3.63 ± 1.18 h, *p* = 0.0153). The children with a height Z-score ≤ −1 had an average RDI of 4.13 ± 3.30, which was higher than those with height Z-scores > −1 (*p* = 0.0041). A significant association was found between BA/CA ratios and sleep duration, where the children with BA/CA ratios > 1.2 experienced longer total sleep duration (8.51 ± 1.28 h) than those with ratios ≤ 1.2 (7.93 ± 1.04 h, *p* = 0.0099). No statistically significant differences in sleep patterns and RDI were observed related to BMI Z-score levels, IGF-1 levels, or the presence or absence of enlarged adenoid tonsils in the posterior nasopharynx. The total sleep duration was significantly longer in the children with moderate or severe OSA (RDI  ≥  5) (8.09 ± 1.09 h) than in the non-OSA children (RDI < 1) (7.24 ± 0.70 h, *p* < 0.0001). However, the shortest deep sleep duration was observed in the children with moderate or severe OSA, a result that was statistically significant (3.07 ± 1.14 h, *p* < 0.0001).

### 3.3. Subgroup Analysis

Age-stratified analyses were conducted to ensure a comprehensive understanding of how sleep patterns vary across the different age groups. Supplementary Table S1 is focused on the children aged 6–9 years. Supplementary Table S2 covers the 10–11 years age group linked to the early puberty stage. Lastly, Supplementary Table S3 is for adolescents aged 12–16 years.

**Figure 1:**
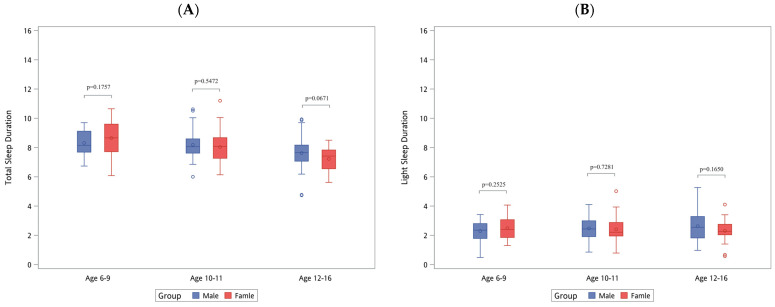

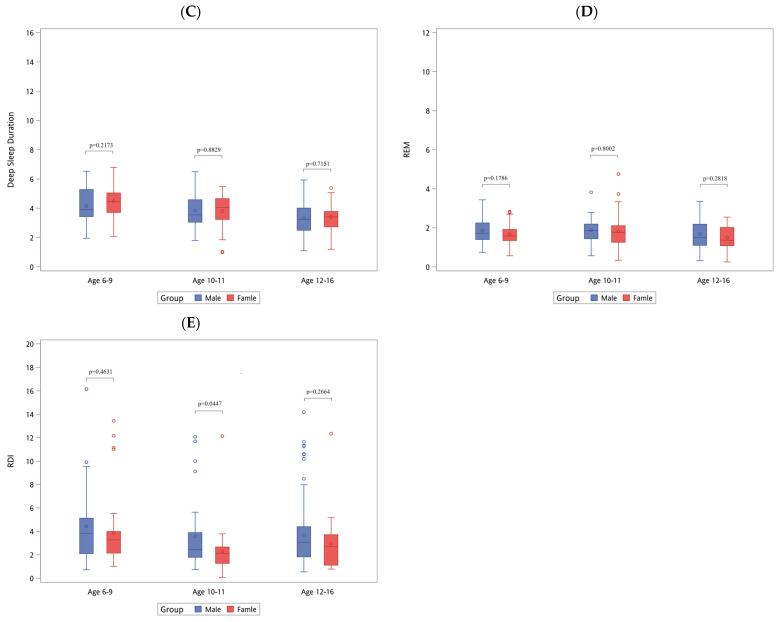
The distribution of sleep patterns and RDI by body sex and age. REM, rapid eye movement; RDI, respiratory disturbance index. (**A**) Total sleep duration (hours). (**B**) Light sleep duration (hours). (**C**) Deep sleep duration (hours). (**D**) Rapid eye movement duration (hours). (**E**) Respiratory disturbance index.

**Figure 2:**
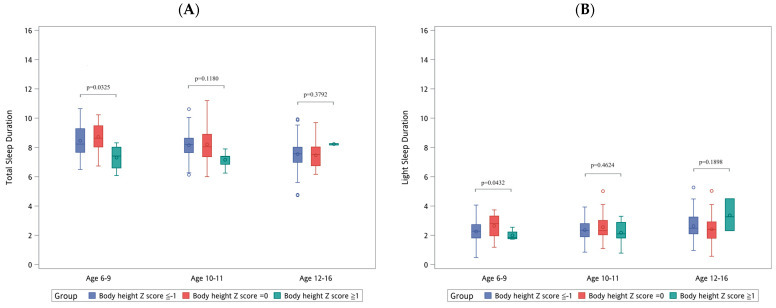

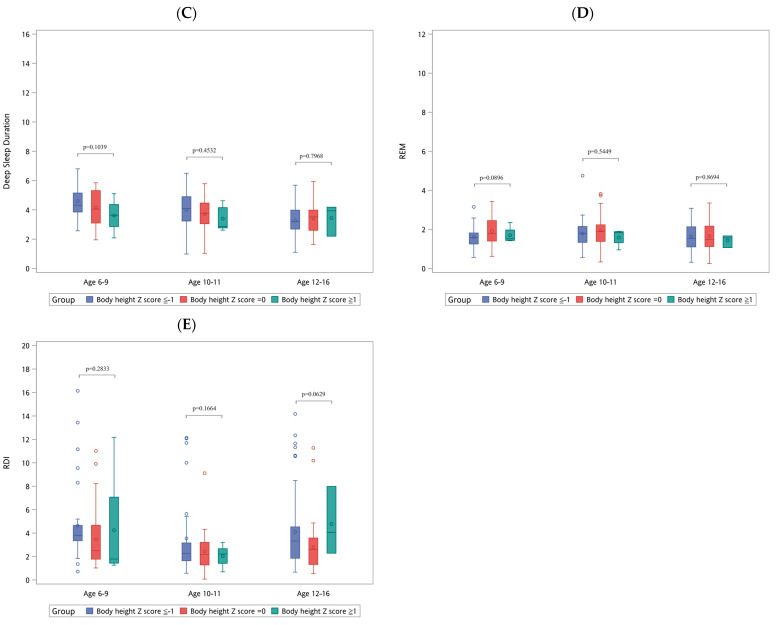
Distribution of sleep patterns and RDI according to body height and age. REM, rapid eye movement; RDI, respiratory disturbance index. (**A**) Total sleep duration (hours). (**B**) Light sleep duration (hours). (**C**) Deep sleep duration (hours). (**D**) Rapid eye movement duration (hours). (**E**) Respiratory disturbance index.

**Figure 3:**
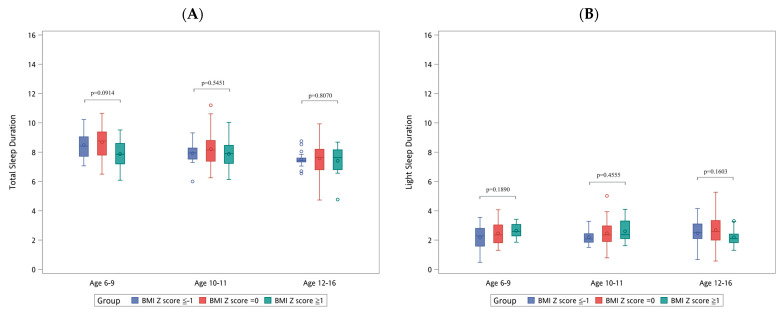

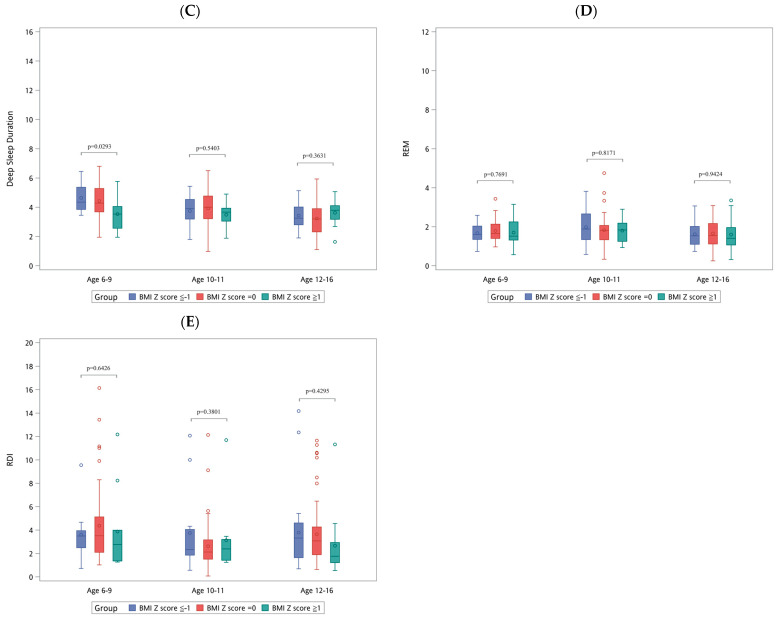
Distribution of sleep patterns and RDI according to BMI and age. REM, rapid eye movement; RDI, respiratory disturbance index; BMI, body mass index. (**A**) Total sleep duration (hours). (**B**) Light sleep duration (hours). (**C**) Deep sleep duration (hours). (**D**) Rapid eye movement duration (hours). (**E**) Respiratory disturbance index.

Figure 1, Figure 2 and Figure 3 illustrate the distribution of sleep patterns and the RDI across different sexes, body heights, and BMI groups, with further stratification according to age group. In Figure 1, which focuses on differences based on sex, Figure 1E highlights that boys within the 10–11 age group exhibited a significantly higher RDI (3.57 ± 3.01) than girls (2.29 ± 1.93), *p* = 0.047. In Figure 2, examining differences based on body height, Figure 2A shows that the children with a body height Z-score of ≥1 in the 6–9 age group had a significantly shorter total sleep duration (7.31 ± 0.95 h), *p* = 0.0325. In Figure 3, which looks at differences by BMI, Figure 3C indicates that the children within the 6–9 age group with a BMI Z-score of ≥1 experienced a significant reduction in deep sleep duration (3.53 ± 1.21 h), *p* = 0.0293.

### 3.4. The Association of Sleep Patterns and Sleep Apnea with Body Height and BMI in Children

Table 3 summarizes the outcomes from the multiple stepwise linear regression analysis for investigating the factors associated with body height among children. In Model 1, focusing on body height as the dependent variable, age per year increment emerged as a significant predictor (beta = 5.19, *p* < 0.0001), with an R^2^ value of 0.769. When slight sleep duration, deep sleep duration, REM sleep duration, and the RDI were added in Model 2, age (beta = 4.89, *p* < 0.0001) remained a significant predictor, and RDI (beta = −0.54, *p* = 0.0022) was significantly negatively associated with body height, enhancing the model R-squared to 0.780.

Table 4 summarizes the outcomes from the multiple stepwise linear regression analysis for investigating the factors associated with BMI among children. Model 3 revealed that age per year increment significantly increases BMI (beta = 3.68, *p* < 0.0001), with an R^2^ of 0.556. When adding the sleep variables in Model 4, age was still a significant factor (beta = 3.33, *p* < 0.0001), and deep sleep duration per hour increment (beta = −0.02, *p* = 0.0371) was significantly negatively associated with BMI, with a slightly improved model fit as indicated by an R^2^ of 0.570.

## 4. Discussion 

The findings of this study revealed there are relationships between age, sex, sleep patterns, and sleep apnea in children with self-perceived short stature. Notably, adolescents aged 12–16 exhibited the shortest overall sleep duration and the least amount of deep sleep compared to all the other examined age groups. Furthermore, girls experienced significantly longer periods of deep sleep than boys. Significant correlations were also identified between age and the RDI as well as between age and body height and between age and BMI. However, no significant correlation was observed between sleep variables and BMI. In future research, it is crucial to devise and rigorously assess interventions tailored to the unique needs and challenges faced by children with self-perceived short stature that take into account their age, sex, and specific endocrine conditions.

Children’s sleep patterns undergo dynamic transformations as they progress through the various developmental stages from infancy to adolescence. Initially, sleep is distributed across multiple periods throughout the day and night, reflecting the polyphasic sleep patterns typical in infants. As children grow, these patterns gradually consolidate into a more monophasic sleep cycle, which is typically characterized by longer nighttime sleep and the reduction or elimination of daytime napping [23,24,25,26]. A significant transition occurs during adolescence, when hormonal changes lead to a phase delay in the sleep–wake cycle that results in delayed sleep onset at night and difficulties with morning awakenings [27,28,29,30,31]. These changes can create a mismatch between adolescents’ biological sleep patterns and societal demands, such as early school start times, potentially leading to chronic sleep deprivation. Adequate sleep duration is essential for optimal growth and development in children, as insufficient sleep has been linked to increased risks of cognitive deficits and physical health issues [12,32]. According to the publications by the AASM in 2016, the American Academy of Pediatrics recommends that school-aged children (6–12 years) should be getting 9–12 h of sleep per 24 h period, while teenagers (13–18 years) should get 8–10 h of sleep daily [11].

During the course of this study, the outbreak of the COVID-19 pandemic led to frequent school closures, with children compelled to adapt to home-based learning environments. In Taiwan, where schools typically start at 8 a.m., our survey revealed that the average sleep duration for this age group was 7.53 ± 0.93 h. We found that adolescents aged 12–17 experienced significantly shorter sleep durations than other age groups, similar to previous study findings. However, it is important to note that our study predominantly involved patients attending a pediatric endocrinology clinic for self-perceived short stature. This specific participant selection might have resulted in an overestimation of sleep duration among children in this particular age group. Consequently, it is imperative in future research to explore potential differences in sleep duration between children receiving treatment for such conditions and their untreated peers.

Several studies have demonstrated that sleep patterns vary according to gender [28,29,30,31,33]. PSG studies have shown that women generally have higher sleep quality than men [34,35]. In their study, Goel et al. [35] highlighted significant differences in sleep characteristics between genders among young, healthy participants undergoing PSG. Compared to men, women were observed to have longer total sleep times, fewer sleep interruptions, shorter latency to sleep onset, quicker transitions to the N1 and N2 sleep stages, and higher sleep efficiency. Consistent with these prior findings [35,36], we also found in our study that girls had longer durations of deep sleep than boys. Dékány et al. [22] found that OSA is also more frequent in men and boys, which may contribute to girls having a comparatively longer duration of deep sleep.

During puberty, significant changes in sleep patterns are correlated with increased levels of sex hormones, such as estrogen and testosterone [27,33], which are linked to a reduction in slow-wave sleep—the deepest stage of sleep [33]. This decrease in slow-wave sleep may reflect the physiological adjustments and brain reorganization that occur during maturation [27,34,35,37]. As observed by Jessen et al. [38], girls experiencing premature pubarche tend to go to sleep later. Additionally, an elevation in sex hormone levels can lead to sleep-related issues, including difficulties in falling asleep, frequent awakenings, and reduced sleep efficiency [27,39,40]. These issues may result from the direct effects of sex hormones on the nervous system or, indirectly, from hormonal fluctuations affecting emotional and stress responses [39]. Our study found that children with BA/CA ratios above 1.2 had significantly longer sleep durations than those with ratios of 1.2 or less. However, we did not find a significant association between IGF-1 and sleep patterns or sleep apnea characteristics. This may be due to the specific population examined in this study, which consists of children with self-perceived short stature.

The BA/CA ratio is a biomarker for evaluating children’s growth and development, where a bone age surpassing the chronological age may indicate accelerated growth or the earlier-than-usual onset of puberty [41,42]. Our findings indicate that children with a BA/CA ratio above 1.2 tend to experience longer sleep durations. The reasons behind this observation are not fully understood and merit further investigation. One potential explanation is that these children may experience reduced sleep quality, requiring longer sleep durations to compensate for this lack of restorative sleep. Nonetheless, the precise factors that may influence sleep quality in this context, such as hormonal fluctuations or alterations in sleep patterns, are yet to be determined. Future research should aim to comprehensively evaluate sleep quality indicators, including sleep disturbances, the distribution of sleep stages, and daytime functionality, to clarify the link between advanced bone age and sleep quality. Longitudinal studies would also be valuable in monitoring how sleep patterns and quality evolve as these children grow older. The confirmation of links between advanced bone age and compromised sleep quality would pave the way for designing customized sleep interventions for this demographic, thereby enhancing their sleep and mitigating possible adverse effects on their health and development.

Previous studies have identified obstructive sleep apnea (OSA) as significantly more prevalent in children under the age of 10 [43], with adenoidal, tonsillar, and adenoid hypertrophy being the primary causes [44,45,46]. These conditions lead to upper airway obstruction due to the relatively larger size of these lymphoid tissues in young children, increasing the likelihood of airway obstruction during sleep [43,44,45,46]. As children develop, these tissues typically regress, resulting in a decreased prevalence of OSA post puberty. In addition to lymphoid tissue hypertrophy, the craniofacial anatomy, including bony structures, plays a crucial role in the risk of OSA, which can explain why symptoms improve as children develop. Another significant risk factor for pediatric OSA is obesity, including the accumulation of fat near the upper airways. The thickness of adipose tissue around the upper airways, as analyzed by artificial intelligence, was found to be significantly correlated with BMI and can predict OSA with high precision [47]. Children with OSA have an increased risk of endocrine, nutritional, metabolic, respiratory, skin, and musculoskeletal disorders [43,44,45,46]. Our study underscores an increased risk of sleep-related breathing disorders among younger children and particularly those with shorter stature, emphasizing the need for healthcare professionals and parents to be vigilant about the potential risks of OSA. It is critical to promptly evaluate and treat OSA symptoms, especially in younger children with the aforementioned anatomical predispositions. Treatment options for pediatric OSA include adenotonsillectomy, continuous positive airway pressure therapy, weight management, and orthodontic interventions. Early diagnosis and appropriate management can enhance sleep quality in children and may prevent further health complications.

While insufficient sleep has been extensively linked to an increased risk of obesity and the potential inhibition of linear growth during adolescence through previous research, the specific effects of sleep duration on stature development remain poorly defined [48,49,50]. This gap underscores the need for thorough investigations into the complex relationships between various sleep metrics and the longitudinal trajectory of height increase [48]. In our study, linear regression models demonstrated limited explanatory power (R^2^ ranging from 0.556 to 0.780), revealing a significant amount of variance in height and BMI unexplained. Furthermore, numerous sleep architecture parameters, including light sleep, slow-wave sleep, and REM sleep duration, were not significant predictors of anthropometric outcomes, except for a slight inverse relationship found between slow-wave sleep and BMI. The cross-sectional design of our study limits the ability to draw causal conclusions between sleep patterns and anthropometric measurements. Additionally, potential confounders such as dietary habits, physical activity levels, and family characteristics were not considered in the models, which could bias the results. Although our study highlights some associations between sleep architecture and anthropometry in children, its observational nature and the limitations of the modeling approach necessitate further longitudinal or interventional research. Such studies should incorporate comprehensive covariate data to more precisely elucidate the impact of sleep on growth and body composition.

## 5. Limitations

This study has several limitations worth mentioning. First, the sample was sourced from a single pediatric endocrinology clinic, potentially limiting the generalizability of our findings to broader populations. Our findings can only be generalized to children aged 6–16 years in central Taiwan. Future studies could be conducted with a more diverse sample, including participants from multiple clinics and different geographic regions, to enhance the generalizability of our results. Second, while home sleep monitoring devices offer a convenient means to study sleep patterns, they may not capture the full spectrum of sleep quality attributes as comprehensively as PSG, the gold standard for sleep research. The accuracy and reliability of these devices might also differ from those employed in controlled laboratory environments. To mitigate this, the PhysioNet Apnea-ECG Database was utilized to test the validity of the LARGAN Health AI-Tech Sleep Apnea and Sleep Quality Examination System. This database comprises 70 polygraphy recordings collected using Sandman SD20 or equivalent polygraph equipment, all manually scored by certified sleep experts. Apneic events were identified, and the AHI was calculated for each recording. The device demonstrated a sensitivity of 95.0% (95% CI: 83.5 to 98.6), a specificity of 90.0% (95% CI: 74.4 to 96.5), and positive predictive values of 92.7%, thus providing a reasonable level of reliability despite the inherent limitations. Third, the cross-sectional design of our study poses challenges in establishing causal relationships. Although it allows for the identification of correlations between variables, it does not enable the determination of direct causality. Future research could aim to employ longitudinal designs to better understand causal relationships between sleep patterns, demographic factors, and health outcomes. Additionally, the lack of a control group does not allow drawing conclusions on whether the observed sleep parameter distribution is typical of all teenagers and children or only of those with this specific condition. Furthermore, this study was conducted during the COVID-19 pandemic, which disrupted daily school and child care routines, increased child stress, and affected child behavior problems [51]. While we recognize that the pandemic likely influenced our findings, we did not have the means to fully quantify its impact. Future studies could analyze the relationships between sleep patterns, demographic factors, and health outcomes in children with self-perceived short stature in the post COVID-19 pandemic era. Finally, our study did not account for all the possible confounders including stress levels, lifestyle habits, dietary habits, physical activity levels, family characteristics, coexisting diseases, and health conditions that could influence sleep quality. Considering these factors might have allowed for a more detailed understanding of the factors affecting sleep.

## 6. Conclusions

In this study, home sleep monitoring devices were utilized to observe the sleep patterns of children with self-perceived short stature, revealing that total sleep time declines as they age. Furthermore, sex significantly influences the sleep patterns of pubescent children. In our study, we found that girls have a longer duration of deep sleep than boys. This difference may be attributed to the higher prevalence of OSA in boys, which results in more frequently disrupted sleep. Additionally, advanced bone age is correlated with longer total sleep times in both genders, likely reflecting their accelerated growth and development pace. After adjusting for sex and age, a significant association between RDI and body height, as well as an association between deep sleep duration and BMI, were observed. These findings highlight the importance of closely monitoring and addressing the sleep health of children with self-perceived short stature, emphasizing the need for tailored sleep care strategies for this particularly vulnerable group. Given that endocrine disorders can significantly impact growth and development, ensuring the proper sleep health of these children is crucial for mitigating additional health risks and promoting overall well-being. Future research should aim to include a broader range of endocrine conditions and their specific impacts on sleep, and prioritize longitudinal studies with comprehensive data collection to better inform tailored sleep interventions and improve health outcomes for children with self-perceived short stature. It is imperative to deepen our understanding and improve the management of sleep disturbances in children with self-perceived short stature, ultimately aiming to enhance their overall health and quality of life.

## Figures and Tables

**Table 1:** Participant demographics, body measurements, sleep patterns, and sleep apnea characteristics.

Variables	Mean ± SD/n(%)
Total number of participants (N)	238 (100.0%)
Month of measurement	
January–March	23 (9.7%)
April–June	13 (5.5%)
July–September	83 (34.9%)
October–December	119 (50.0%)
Day of week	
Friday–Saturday (weekend night)	204 (85.7%)
Sunday–Thursday (weeknight)	34 (14.3%)
Sex	
Female	101 (42.4%)
Male	137 (57.6%)
Age (Range: 6–16 years)	
6–9 years old	72 (30.3%)
10–11 years old	68 (28.6%)
12–16 years old	98 (41.2%)
Average body height (cm)	140.55 ± 14.59
Body height Z-score	−0.85 ± 1.02
Average BMI (kg/m^2^)	35.84 ± 12.53
BMI Z-score	−0.21 ± 1.26
Bone age to chronological age ratio (BA/CA)	1.00 ± 0.15
Average IGF-1 (ng/mL)	234.91 ± 108.72
IGF-1 Z-score	0.00 ± 0.96
Enlarged adenoid tonsil in posterior nasopharynx	
Absent	151 (63.5%)
Present	87 (36.6%)
Total sleep duration (hours)	7.99 ± 1.08
Light sleep duration (hours)	2.48 ± 0.85
Deep sleep duration (hours)	3.79 ± 1.18
REM sleep duration (hours)	1.73 ± 0.68
Respiratory disturbance index (Range: 0.07–16.14)	3.51 ± 2.87
RDI < 1	19 (8.0%)
1 ≤ RDI < 5	184 (77.3%)
RDI ≥ 5	35 (14.7%)
Sleep latency	
<30 min	139 (58.4%)
≥30 min	99 (41.6%)
Growth hormone therapy	
No	170 (71.2%)
Yes	68 (28.6%)

**Table 2:** Distribution of sleep patterns and respiratory disturbance index according to demographic and body measurement characteristics in children.

	N	Total Sleep (Hours)	Light Sleep (Hours)	Deep Sleep (Hours)	REM (Hours)	RDI
Entire sample	238	7.99 ± 1.08	2.48 ± 0.85	3.79 ± 1.18	1.73 ± 0.68	3.51 ± 2.87
Month of measurement						
APR–SEP (summer)	96	8.10 ± 1.10	2.41 ± 0.73	3.82 ± 1.18	1.87 ± 0.72	3.66 ± 3.06
OCT–DEC, JAN–MAR (winter)	142	7.92 ± 1.07	2.52 ± 0.92	3.76 ± 1.18	1.64 ± 0.64	3.41 ± 2.74
*p*-value		0.2035	0.3098	0.6882	0.0110 *	0.5095
Day of week						
Friday–Saturday (weekend night)	204	7.94 ± 1.05	2.46 ± 0.86	3.77 ± 1.18	1.71 ± 0.68	3.48 ± 2.84
Sunday–Thursday (weeknight)	34	8.28 ± 1.25	2.57 ± 0.80	3.86 ± 1.21	1.86 ± 0.71	3.73 ± 3.06
*p*-value		0.0947	0.5047	0.7053	0.2440	0.6396
Age						
6–9 years old	72	8.51 ± 1.04	2.41 ± 0.72	4.36 ± 1.11	1.75 ± 0.59	4.10 ± 3.04
10–11 years old	68	8.11 ± 1.07	2.44 ± 0.78	3.82 ± 1.19	1.85 ± 0.78	2.90 ± 2.56
12–16 years old	98	7.53 ± 0.93	2.55 ± 0.97	3.34 ± 1.03	1.64 ± 0.67	3.51 ± 2.88
*p*-value		<0.0001 *	0.4937	<0.0001 *	0.1484	0.0457
Sex						
Female	101	8.10 ± 1.20	2.42 ± 0.79	4.00 ± 1.15	1.68 ± 0.69	3.10 ± 2.56
Male	137	7.91 ± 0.99	2.52 ± 0.89	3.63 ± 1.18	1.76 ± 0.68	3.83 ± 3.06
*p*-value		0.1781	0.3694	0.0153 *	0.3952	0.0530
Height Z-score						
≤−1	116	7.99 ± 1.09	2.44 ± 0.83	3.89 ± 1.26	1.67 ± 0.67	4.13 ± 3.30
0	110	8.04 ± 1.10	2.52 ± 0.86	3.71 ± 1.11	1.81 ± 0.71	2.88 ± 2.12
≥1	12	7.48 ± 0.78	2.41 ± 0.97	3.48 ± 0.97	1.58 ± 0.39	3.46 ± 3.34
*p*-value		0.2286	0.7574	0.3629	0.2229	0.0041 *
BMI Z-score						
≤−1	54	7.94 ± 0.84	2.30 ± 0.78	3.92 ± 1.08	1.72 ± 0.63	3.71 ± 2.89
0	147	8.09 ± 1.17	2.55 ± 0.91	3.79 ± 1.26	1.74 ± 0.68	3.54 ± 2.86
≥1	37	7.67 ± 1.01	2.43 ± 0.65	3.56 ± 0.93	1.69 ± 0.78	3.13 ± 2.93
*p*-value		0.1075	0.1651	0.3470	0.8919	0.6290
BA/CA ratios						
≤1.2	212	7.93 ± 1.04	2.47 ± 0.87	3.73 ± 1.18	1.73 ± 0.69	3.60 ± 2.93
>1.2	26	8.51 ± 1.28	2.49 ± 0.69	4.26 ± 1.11	1.76 ± 0.62	2.86 ± 2.27
*p*-value		0.0099 *	0.9127	0.0312 *	0.8197	0.2152
IGF-1						
<0	126	8.08 ± 1.09	2.49 ± 0.88	3.82 ± 1.20	1.77 ± 0.74	3.74 ± 3.06
≥0	112	7.89 ± 1.07	2.46 ± 0.82	3.75 ± 1.16	1.68 ± 0.61	3.26 ± 2.62
*p*-value		0.1900	0.8332	0.6381	0.3103	0.1941
Enlarged adenoid tonsil in posterior nasopharynx						
Absent	151	7.98 (±1.04)	2.49 (±0.75)	3.72 (±1.14)	1.77 (±0.63)	3.67 (±3.06)
Present	87	8.01 (±1.16)	2.45 (±0.99)	3.90 (±1.24)	1.66 (±0.76)	3.25 (±2.50)
*p*-value		0.8245	0.7812	0.2691	0.2606	0.2546
Growth hormone therapy						
No	170	8.01 ± 1.02	2.46 ± 0.78	3.83 ± 1.13	1.73 ± 0.64	3.73 ± 3.17
Yes	68	7.93 ± 1.24	2.52 ± 1.01	3.68 ± 1.30	1.74 ± 0.79	2.98 ± 1.82
*p*-value		0.6317	0.6544	0.3718	0.9353	0.0226 *
RDI						
RDI < 1	19	7.24 ± 0.70	1.56 ± 0.72	4.31 ± 1.31	1.36 ± 0.67	0.71 ± 0.20
1 ≤ RDI < 5	184	8.05 ± 1.09	2.44 ± 0.76	3.87 ± 1.12	1.74 ± 0.67	2.71 ± 1.07
5 ≤ RDI	35	8.09 ± 1.09	3.16 ± 0.82	3.07 ± 1.14	1.85 ± 0.71	9.27 ± 3.04
*p*-value		<0.0001 *	0.4937	<0.0001 *	0.1484	<0.0001 *

REM, rapid eye movement; RDI, respiratory disturbance index; BMI, body mass index; BA, bone age; CA, chronological age; IGF-1, insulin-like growth factor 1. * *p* < 0.05.

**Table 3:** Linear regression model for body height as a dependent factor.

	Dependent Variable: Body Height			
	Model 1		Model 2	
Parameter	Beta coefficients (95% CI)	t value/*p*-value	Beta coefficients (95% CI)	t value/*p*-value
Sex (male)	−0.35 (−2.27, 1.57)	−0.36/0.7186	−0.83 (−2.75, 1.08)	−0.86/0.3927
Age (year)	5.19 (4.80, 5.57) *	26.44/<0.0001	4.89 (4.45, 5.33) *	21.74/<0.0001
Light sleep duration (hour)			0.01 (−0.01, 0.03)	0.84/0.4009
Deep sleep duration (hour)			−0.02 (−0.03, 0.00)	−1.82/0.0708
REM (hour)			−0.01 (−0.04, 0.01)	−1.00/0.3165
RDI			−0.54 (−0.88, −0.20) *	−3.10/0.0022
Model Fit				
R^2^	0.769		0.780	

REM, rapid eye movement; RDI, respiratory disturbance index; CI, confidence interval. Model 1 includes the co-variates of sex (male) and age. Model 2 includes the co-variates of sex (male), age, light sleep duration, deep sleep duration, REM, and RDI. * indicates *p*-value < 0.05. R^2^ = coefficient of determination.

**Table 4:** Linear regression model for BMI as a dependent factor.

	Dependent Variable: BMI			
	Model 3		Model 4	
Parameter	Beta coefficients (95% CI)	t value/*p*-value	Beta coefficients (95% CI)	t value/*p*-value
Sex (male)	−1.70 (−3.98, 0.58)	−1.47/0.1437	−2.16 (−4.45, 0.14)	−1.85/0.0661
Age (year)	3.68 (3.22, 4.14) *	15.80/<0.0001	3.33 (2.80, 3.86) *	12.34/<0.0001
Light sleep duration (hour)			−0.00 (−0.03, 0.03)	−0.05/0.9629
Deep sleep duration (hour)			−0.02 (−0.04, −0.00) *	−2.10/0.0371
REM (hour)			−0.03 (−0.06, 0.00)	−1.89/0.0605
RDI			−0.40 (−0.81, 0.01)	−1.91/0.0570
Model Fit				
R^2^	0.556		0.570	

REM, rapid eye movement; RDI, respiratory disturbance index; BMI, body mass index; CI, confidence interval. Model 3 includes the co-variates of sex (male) and age. Model 4 includes the co-variates of sex (male), age, light sleep duration, deep sleep duration, REM, and RDI. * indicates *p*-value < 0.05. R^2^ = coefficient of determination.

## Data Availability

The dataset cannot be shared publicly due to concerns about patient confidentiality and privacy. Inquiries regarding the data can be directed to the corresponding author.

## Supplementary Materials

The following supporting information can be downloaded at: https://www.mdpi.com/article/10.3390/diagnostics14151675/s1.
