# Supplementary material for: Association of Sleep Patterns and Respiratory Disturbance Index with Physiological Parameters in Pediatric Patients with Self-Perceived Short Stature
Source: Diagnostics (Basel). 2024 Aug 2;14(15):1675. doi: 10.3390/diagnostics14151675 (PMC11311679; doi:10.3390/diagnostics14151675)
Supplement: Supplementary file 1 [file diagnostics-14-01675-s001.zip › diagnostics-3068995-supplementary.pdf]

PREPARATION BEFORE GOING TO BED	
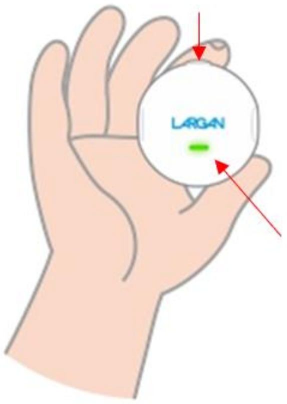	1. Pressing the button on top of the ECG, it's ready to go if you see either **GREEN** or **BLUE** light. If the light appears to be **YELLOW** or **RED**, please return the ECG back to us.
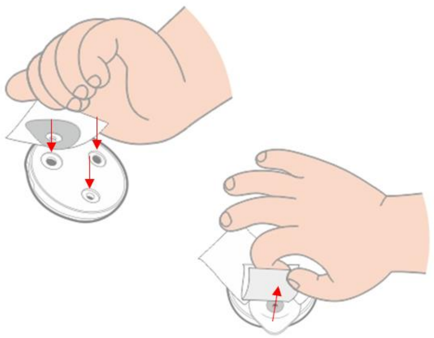	2. Attaching the 3 stickers onto ECG then tear the released paper off.
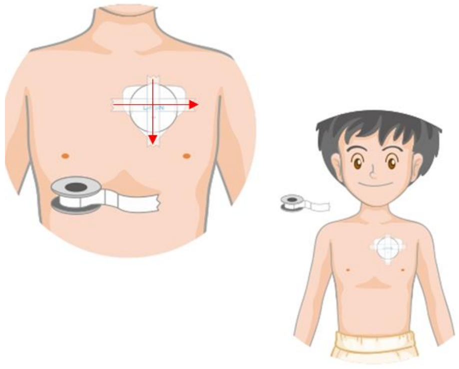	3. Putting the ECG on (making sure **the BUTTON is on TOP**) by your heart with surgical tapes crossing with each other on tape of the ECG tightly.
PROCEDURES AFTER WAKING UP	
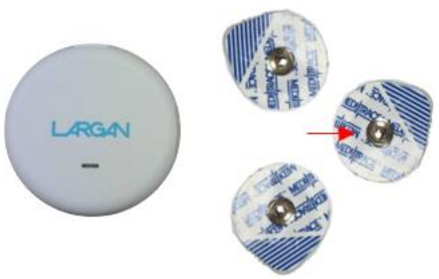	4. Having the ECG removed as soon as you wake up.

Supplementary Figure S1 Instructions of wearing LARGAN ECG

STROBE Statement—Checklist of items that should be included in reports of ***cross-sectional studies***

	Item No	Recommendation	Page Number
**Title and abstract**	1	(*a*) Indicate the study's design with a commonly used term in the title or the abstract	1
		(*b*) Provide in the abstract an informative and balanced summary of what was done and what was found	2
**Introduction**			
Background/rationale	2	Explain the scientific background and rationale for the investigation being reported	3
Objectives	3	State specific objectives, including any prespecified hypotheses	3-4
**Methods**			
Study design	4	Present key elements of study design early in the paper	4
Setting	5	Describe the setting, locations, and relevant dates, including periods of recruitment, exposure, follow-up, and data collection	4
Participants	6	(*a*) Give the eligibility criteria, and the sources and methods of selection of participants	4
Variables	7	Clearly define all outcomes, exposures, predictors, potential confounders, and effect modifiers. Give diagnostic criteria, if applicable	4-5
Data sources/ measurement	8*	For each variable of interest, give sources of data and details of methods of assessment (measurement). Describe comparability of assessment methods if there is more than one group	4-5
Bias	9	Describe any efforts to address potential sources of bias	17
Study size	10	Explain how the study size was arrived at	4
Quantitative variables	11	Explain how quantitative variables were handled in the analyses. If applicable, describe which groupings were chosen and why	4-5
Statistical methods	12	(*a*) Describe all statistical methods, including those used to control for confounding	5-6
		(*b*) Describe any methods used to examine subgroups and interactions	5-6
		(*c*) Explain how missing data were addressed	NA
		(*d*) If applicable, describe analytical methods taking account of sampling strategy	4
		(*e*) Describe any sensitivity analyses	5-6
**Results**			
Participants	13*	(a) Report numbers of individuals at each stage of study—eg numbers potentially eligible, examined for eligibility, confirmed eligible, included in the study, completing follow-up, and analysed	6
		(b) Give reasons for non-participation at each stage	4
		(c) Consider use of a flow diagram	NA
Descriptive data	14*	(a) Give characteristics of study participants (eg demographic, clinical,	6, Table

		social) and information on exposures and potential confounders	1
		(b) Indicate number of participants with missing data for each variable of interest	4
Outcome data	15*	Report numbers of outcome events or summary measures	6-10<br>Table 2<br>Table 3
Main results	16	(*a*) Give unadjusted estimates and, if applicable, confounder-adjusted estimates and their precision (eg, 95% confidence interval). Make clear which confounders were adjusted for and why they were included	6-10<br>Table 2<br>Table 3
		(*b*) Report category boundaries when continuous variables were categorized	6-10<br>Table 2<br>Table 3
		(*c*) If relevant, consider translating estimates of relative risk into absolute risk for a meaningful time period	6-10<br>Table 2<br>Table 3
Other analyses	17	Report other analyses done—eg analyses of subgroups and interactions, and sensitivity analyses	10<br>Figure 1-<br>Figure 3
**Discussion**			
Key results	18	Summarise key results with reference to study objectives	15
Limitations	19	Discuss limitations of the study, taking into account sources of potential bias or imprecision. Discuss both direction and magnitude of any potential bias	17
Interpretation	20	Give a cautious overall interpretation of results considering objectives, limitations, multiplicity of analyses, results from similar studies, and other relevant evidence	17
Generalisability	21	Discuss the generalisability (external validity) of the study results	15-17
**Other information**			
Funding	22	Give the source of funding and the role of the funders for the present study and, if applicable, for the original study on which the present article is based	18

*Give information separately for exposed and unexposed groups.

Note: An Explanation and Elaboration article discusses each checklist item and gives methodological background and published examples of transparent reporting. The STROBE checklist is best used in conjunction with this article (freely available on the Web sites of PLoS Medicine at http://www.plosmedicine.org/, Annals of Internal Medicine at http://www.annals.org/, and Epidemiology at http://www.epidem.com/). Information on the STROBE Initiative is available at www.strobe-statement.org.

Supplementary Table S1 Distribution of Sleep Pattern and Respiratory Disturbance Index by Demographic and Body Measurements Characteristics in the 6-9-Year-Old Group

Basic Characteristics	N	Total sleep (hours)	Light sleep (hours)	Deep sleep (hours)	REM (hours)	RDI
Age 6-9	72	8.51±1.04	2.41±0.72	4.36±1.11	1.75±0.59	4.10±3.04
Gender						
Female	42	8.65±1.15	2.49±0.73	4.49±1.01	1.67±0.54	3.88±2.89
Male	30	8.31±0.83	2.29±0.70	4.17±1.23	1.86±0.64	4.41±3.27
p-value		0.1757	0.2525	0.2173	0.1786	0.4631
Height Z score						
≦-1	37	8.46±1.05	2.26±0.67	4.60±1.06	1.61±0.51	4.63±3.18
0	31	8.72±0.95	2.64±0.76	4.16±1.12	1.92±0.66	3.45±2.49
≧1	4	7.31±0.95	2.00±0.38	3.61±1.23	1.70±0.44	4.25±5.29
p-value		0.0325	0.0432	0.1039	0.0896	0.2833
BMI Z score						
≦-1	19	8.49±0.89	2.17±0.77	4.64±0.94	1.67±0.47	3.60±1.74
0	43	8.67±1.07	2.46±0.72	4.42±1.09	1.79±0.58	4.37±3.38
≧1	10	7.87±1.04	2.64±0.53	3.53±1.21	1.70±0.85	3.88±3.57
p-value		0.0914	0.1890	0.0293	0.7691	0.6426
BA/CA						
≦1.2	56	8.43±0.95	2.35±0.74	4.36±1.12	1.72±0.56	4.36±3.11
>1.2	16	8.79±1.30	2.61±0.64	4.36±1.14	1.83±0.70	3.20±2.67
p-value		0.2236	0.2182	0.9948	0.532	0.1833
IGF-1						
<0	35	8.61±1.06	2.31±0.74	4.53±1.03	1.77±0.62	3.73±2.22
≧0	37	8.41±1.02	2.50±0.71	4.19±1.17	1.73±0.56	4.44±3.65
p-value		0.4254	0.2872	0.1955	0.7856	0.3194

The data includes the mean and standard deviation values for different measures of sleep.

REM, Rapid Eye Movement; RDI, Respiratory Disturbance Index; BMI, Body Mass Index; BA, Bone Age; CA, chronological age; IGF-1, Insulin-like Growth Factor 1.

Supplementary Table S2 Distribution of Sleep Pattern and Respiratory Disturbance Index by Demographic and Body Measurements Characteristics in the 10-11-Year-Old Group

	N	Total sleep (hours)	Light sleep (hours)	Deep sleep (hours)	REM (hours)	RDI
10-11 Years Old	68	8.11±1.07	2.44±0.78	3.82±1.19	1.85±0.78	2.90±2.56
Gender						
Female	36	8.03±1.14	2.41±0.84	3.81±1.19	1.82±0.87	2.29±1.93
Male	32	8.19±1.00	2.47±0.72	3.85±1.22	1.87±0.68	3.57±3.01
p-value		0.5472	0.7281	0.8829	0.8002	0.0447
Height Z score						
≦-1	30	8.15±0.98	2.36±0.67	4.01±1.28	1.79±0.82	3.55±3.36
0	33	8.21±1.16	2.55±0.86	3.72±1.15	1.94±0.79	2.43±1.62
≧1	5	7.16±0.63	2.18±0.98	3.40±0.91	1.58±0.43	2.04±1.00
p-value		0.1180	0.4624	0.4532	0.5449	0.1664
BMI Z score						
≦-1	12	7.92±0.86	2.20±0.50	3.75±1.07	1.98±0.89	3.75±3.59
0	45	8.21±1.14	2.46±0.85	3.93±1.30	1.82±0.80	2.61±2.12
≧1	11	7.88±1.02	2.59±0.76	3.49±0.84	1.80±0.63	3.13±2.93
p-value		0.5451	0.4555	0.5403	0.8171	0.3801
BA_CA						
≦1.2	59	8.11±1.06	2.47±0.79	3.77±1.21	1.87±0.82	3.02±2.69
>1.2	9	8.06±1.24	2.21±0.72	4.19±1.12	1.67±0.49	2.07±1.19
p-value		0.8970	0.3566	0.3347	0.4665	0.0828
IGF-1						
<0	39	8.30±1.11	2.49±0.72	3.82±1.11	1.99±0.79	3.16±3.01
≧0	29	7.85±0.98	2.36±0.86	3.84±1.32	1.65±0.74	2.54±1.77
p-value		0.0880	0.4903	0.9478	0.0803	0.2851

The data includes the mean and standard deviation values for different measures of sleep.

REM, Rapid Eye Movement; RDI, Respiratory Disturbance Index; BMI, Body Mass Index; BA, Bone Age; CA, chronological age; IGF-1, Insulin-like Growth Factor 1.

Supplementary Table S3 Distribution of Sleep Pattern and Respiratory Disturbance Index by Demographic and Body Measurements Characteristics in the 12-16-Year-Old Group

Variables	N	Total sleep (hours)	Light sleep (hours)	Deep sleep (hours)	REM (hours)	RDI
Age 12-16	98	7.53±0.93	2.55±0.97	3.34±1.03	1.64±0.67	3.51±2.88
Gender						
Female	23	7.22±0.76	2.31±0.82	3.41±0.99	1.50±0.58	2.93±2.45
Male	75	7.62±0.96	2.63±1.00	3.32±1.05	1.68±0.69	3.69±2.99
p-value	-	0.0671	0.165	0.7151	0.2818	0.2664
Height Z score						
≤-1	49	7.54±1.03	2.63±0.98	3.27±1.08	1.64±0.69	4.11±3.36
0	46	7.47±0.82	2.42±0.93	3.41±0.99	1.64±0.67	2.80±2.12
≥1	3	8.23±0.06	3.36±1.09	3.44±1.08	1.43±0.32	4.78±2.92
p-value	-	0.3792	0.1898	0.7968	0.8694	0.0629
BMI Z score						
≤-1	23	7.50±0.49	2.46±0.90	3.42±0.90	1.62±0.58	3.79±3.34
0	59	7.57±1.04	2.69±1.06	3.23±1.11	1.65±0.66	3.64±2.77
≥1	16	7.40±0.97	2.18±0.59	3.62±0.84	1.60±0.85	2.66±2.58
p-value	-	0.807	0.1603	0.3631	0.9424	0.4295
BA_CA						
≤1.2	97	7.52±0.93	2.55±0.97	3.34±1.04	1.64±0.67	3.51±2.89
>1.2	1	NA	NA	NA	NA	NA
p-value	-	NA	NA	NA	NA	NA
IGF-1 Z score						
<0	52	7.55±0.86	2.60±1.05	3.34±1.13	1.61±0.75	4.18±3.54
≥0	46	7.50±1.00	2.50±0.88	3.34±0.91	1.66±0.57	2.76±1.62
p-value	-	0.7851	0.6298	0.9705	0.7050	0.0110

The data includes the mean and standard deviation values for different measures of sleep.

REM, Rapid Eye Movement; RDI, Respiratory Disturbance Index; BMI, Body Mass Index; BA, Bone Age; CA, chronological age; IGF-1, Insulin-like Growth Factor 1.
